# Fascia Tympanoplasty: A Tertiary Center Experience

**DOI:** 10.7759/cureus.85237

**Published:** 2025-06-02

**Authors:** Moayyad Malas, Raghad S Alzahrani, Fuad A Sindi, Ali S Alsudais, Ibrahim S Binrabaa, Saleh A Al-Zahrani, Haya Alsubaie

**Affiliations:** 1 Department of Otolaryngology, East Jeddah Hospital, Ministry of Health, Jeddah, SAU; 2 College of Medicine, King Saud bin Abdulaziz University for Health Sciences, Jeddah, SAU; 3 Department of Research, King Abdullah International Medical Research Center, Jeddah, SAU; 4 Department of Otolaryngology, King Abdulaziz Medical City, Jeddah, SAU

**Keywords:** hearing improvement, perforation, residual, surgical outcomes, tympanoplasty

## Abstract

Background: Tympanoplasty is a surgical procedure performed to repair a perforated tympanic membrane and restore hearing function. Understanding the risk factors associated with graft failure or recurrence is crucial for optimizing surgical outcomes. This retrospective study aimed to analyze the outcomes of tympanoplasty and identify potential risk factors using a tertiary center experience.

Methods: A retrospective cohort study was conducted using medical records from King Abdulaziz Medical City in Jeddah. Patients who underwent tympanoplasty surgery between 2017 and 2023 were included. Demographic characteristics, clinical factors, and surgical outcomes were analyzed using descriptive statistics and statistical tests.

Results: Among 71 fascia tympanoplasty procedures, the success rate was 83.1%. Among 38 patients with post-operative hearing assessment, 57.9% showed improved hearing gap. Age at the time of surgery and post-operative decibel levels were significantly associated with hearing improvement. Post-operative residual perforation occurred in 16.9% of patients and was associated with changes in hearing gap. Most patients had successful graft uptake. The procedure was safe, with a low incidence of complications.

Conclusion: Fascia tympanoplasty demonstrated favorable outcomes in terms of success rate, hearing improvement, graft uptake, and safety. Age and closure of residual perforation were identified as significant factors influencing the surgical outcome. However, the study had limitations such as its retrospective design and the small sample size. Further research with larger sample sizes and prospective designs is needed to confirm these findings and optimize the management of tympanic membrane perforations.

## Introduction

Tympanoplasty is a surgical procedure that aims to repair a perforated tympanic membrane, eradicate a middle ear infection, and restore auditory functions. The surgery involves grafting the tympanic membrane with or without reconstruction of the middle ear bones [1].

Several classification systems have been developed to describe and standardize tympanoplasty techniques based on the extent of middle ear damage and the method of reconstruction. The earliest of these was the Wullstein classification introduced in 1956 [2], which categorized the procedure into five types. Subsequently, multiple modifications were proposed to refine and expand this system. In 1971, Farrior [3] sub-classified the third and fifth types, followed by Bellucci’s [4] dual classification approach in 1973, which considered both pre- and post-operative conditions. In 1974, Pratt [5] further modified the Wullstein and Farrior systems by adding a sixth type. Later, in 2005, Nadol and McKenna [6] updated the classification by removing types IV and V and subdividing type III into three subcategories. Most recently, Kim [7] introduced additional revisions, including the addition of type 0 and the elimination of type V [8].

Over the years, various techniques have been attempted to achieve better outcomes. For example, over-under tympanoplasty combines two techniques, overlay and underlay, by placing the graft over the malleus and under the annulus [9]. This hybrid procedure overcomes the disadvantages of the two techniques by preventing anterior blunting and middle ear space reduction. It is also suited for ossicular reconstruction. Microclip tympanoplasty is another technique where one millimeter of stainless-steel wire is used to hold the graft in position [10]. Finally, the newest technique is endoscopic tympanoplasty. The surgery is performed through an incision in the ear canal. It is less invasive than the microscopic methods that use endaural or postauricular approaches [1]. Moreover, it provides a wider view of the middle ear, takes less operation time, and produces less post-operative pain [11].

Furthermore, autologous and alloplastic materials are the two types of grafts used in tympanoplasty. Autologous materials are grafted from the patient’s body such as temporal fascia, fascia lata, periosteum, skin canal, and conchal perichondrium. Therefore, they are inexpensive, biocompatible, and available [12].

On the other hand, commonly used alloplastic grafts, such as acellular dermal matrix, paper, and absorbable gelatin sponge, are believed to offer certain advantages, including faster healing, minimal scarring, reduced pain, quicker recovery, and lower infection risk, although these benefits remain unproven in clinical practice [13]. In contrast, autologous grafts, most notably temporalis fascia, are widely favored due to their accessibility, biocompatibility, and long-standing use in tympanoplasty, with cartilage grafts often serving as an alternative [1]. As the graft type is considered a critical factor influencing surgical outcomes, its role warrants consideration when evaluating potential contributors to graft success or failure in tympanoplasty.

The success of the tympanoplasty procedure can be evaluated anatomically and functionally. An intact dry tympanic membrane by six months post-operation is considered an anatomical success, whereas functional success is associated with hearing improvement and air-bone gap closure to ≤20 dB. Several factors influence the success rate. The patient's age, size and site of membrane perforation, the status of middle ossicles and mastoid, operative technique, type of graft, and the surgical experience are important variables affecting the success rate [14]. A study evaluated the influence of some prognostic factors on the success of type 1 tympanoplasty in pediatric patients with chronic otitis media. The success rate was 86.3%, which is consistent with rates based on the literature. Factors such as age, size and site of perforation, and the surgical technique had a statistically insignificant effect on the outcome. Other factors, such as contralateral ear status and graft material, had a significant effect on the outcome. The literature attributes the surgery failure at an early age to insufficient pharyngotympanic tube function and a weak immune system causing recurrent respiratory tract infections [15]. In contrast, a similar study stated that the correlation between the function of the Eustachian tube and the success rate was insignificant [16]. However, the results of a systematic review on the influence of several factors on tympanoplasty success stated that age, size of perforation, the status of the opposite ear, and surgical experience were statistically significant factors [17].

Furthermore, the selected type of graft is an important effector to the success of the procedure. The most common choice is temporalis fascia as it is near the surgery site and easy to harvest. Another common choice is a cartilage graft collected from the autologous concha or tragus. In comparison, anatomical success was higher in cartilage grafts, whereas anatomical success did not differ. Also, the temporalis fascia grafts have substandard stability features because they contain connective fibrous tissue with irregular elastic fibers. On the other hand, cartilage grafts provide strength, durability, protection against infection, and resistance against low blood supply and high pressure. Therefore, they are more suitable for revision tympanoplasty, reconstruction of a previously failed tympanoplasty [14]. Moreover, graft take-up is significantly higher in dry ears. Other statistically significant factors affecting graft viability are the site of perforation, surgical technique, age, smoking, contralateral ear status, and age [18].

Gaining a comprehensive understanding of the risk factors linked to graft failure or recurrence is of utmost importance when it comes to maximizing the success of tympanoplasty. In this retrospective study, our primary objective was to thoroughly analyze the outcomes of tympanoplasty and to identify potential risk factors contributing to unfavorable results. To achieve this, we conducted an in-depth investigation based on the extensive experience of a tertiary center.

## Materials and methods

Study design and setting

This retrospective cohort study was conducted at King Abdulaziz Medical City (KAMC), a tertiary referral hospital located in Jeddah, in the western region of Saudi Arabia. The study included all patients who underwent tympanoplasty surgery at KAMC between January 2017 and April 2023. The total sample comprised the entire population of eligible cases during this period, estimated to be approximately 80 patients.

Inclusion criteria consisted of all patients who underwent tympanoplasty, regardless of surgical technique or indication. The only exclusion criterion was a confirmed diagnosis of cholesteatoma, either pre-operatively or post-operatively, due to its distinct pathophysiology, higher recurrence rate, and potential to confound outcome assessment. No additional exclusions (e.g., revision cases, ossiculoplasty) were applied in order to maintain a comprehensive and real-world representation of tympanoplasty outcomes.

A non-probability consecutive (inclusive) sampling method was employed, whereby all eligible patients meeting the criteria within the study period were included. This approach was chosen to ensure full coverage of available cases and to minimize selection bias associated with more restrictive or randomized sampling techniques.

Data collection

Data were extracted from the hospital’s electronic medical record system, BestCare, and organized in a structured Excel spreadsheet. Collected variables included patient demographics (age and gender), surgical history (previous ear surgeries), comorbidities (e.g., diabetes, hypertension, dyslipidemia, anemia, hypothyroidism, hypercholesterolemia, asthma), affected ear side, and the presence of contralateral tympanic membrane perforation.

Pre-operative determinants included the size and location of the tympanic membrane (TM) perforation, audiogram results, tympanometry, CT scan findings (to assess for cholesteatoma or ossicular erosion), and pre-operative hearing thresholds. The TM perforation size was classified as pinhole (<20%), small (20-60%), and large (>60%) perforations. The perforation location was described as anterior, posterior, or central/inferior. Hearing loss was categorized as conductive, sensorineural (SNHL), or mixed based on audiometric criteria, with normal thresholds defined as <20 dB, and mild to moderate conductive loss indicated by air-bone gaps of 20-40 dB and 41-60 dB, respectively.

Post-operative outcomes were assessed by comparing pre- and post-operative hearing thresholds (in decibels), evaluating the presence of residual or recurrent perforation, and documenting the need for revision surgery. Residual perforation referred to immediate post-operative graft failure, while recurrence was defined as new perforation after initial healing. Additionally, post-operative complications (e.g., infection, pain, graft necrosis, numbness, tinnitus, and reduced hearing) were recorded. When available, specific infections, such as otitis media, otitis externa, Methicillin-resistant Staphylococcus aureus (MRSA)-related infections, or overgrowth of skin flora, were documented.

Statistical analysis

Descriptive analysis presented continuous variables as mean, standard deviation (SD), and interquartile range, while categorical variables were expressed as frequencies, percentages, and graphical representations. For statistical analysis, JMP 2.0 software was utilized for all comparisons. A t-test was conducted to evaluate the equality of means for normally distributed continuous variables. The chi-square test was used to compare categorical data. The Mann-Whitney test, a non-parametric alternative, was employed for continuous variables that did not meet the assumption of normality. This ensured more accurate comparisons when data distribution was skewed or sample sizes were limited. The confidence interval was set at 95%, and a p-value less than 0.05 (p<0.05) was considered statistically significant.

## Results

The study included a total of 71 patients who underwent tympanoplasty. Table 1 provides a comprehensive overview of the characteristics of these patients. The average age of the study cohort was 32.39 years (SD = 12.75), ranging from 18 to 65 years. Among the patients, 39 (54.9%) were female. Only two (2.8%) patients had undergone multiple surgeries in the same ear. Regarding bilateral involvement, nine (12.7%) patients require surgery in both ears at different times, as shown in Table 1.

**Table 1:** Participants’ characteristics. CHL: conductive hearing loss; HL: hearing loss; SNHL: sensorineural hearing loss; DM: diabetes mellitus; HTN: hypertension

Characteristic		N	%	Missing
Age, mean (SD)		32.39 (12.75)		n=0
Gender	Male	32	45.1%	n=0
	Female	39	54.9%	
More than one surgery in the same ear	No	69	97.2%	n=0
	Yes	2	2.8%	
Surgery in both ears at different times	No	62	87.3%	n=0
	Yes	9	12.7%	
DM	No	66	93.0%	n=0
	Yes	5	7.0%	
HTN	No	68	95.8%	n=0
	Yes	3	4.2%	
Other comorbidities	Yes	25	35.2%	n=0
	None	46	64.8%	
Contralateral tympanic membrane perforation	No	44	62.0%	n=0
	Yes	27	38.0%	
Site of ear	Left	33	46.5%	n=0
	Right	38	53.5%	
Size of perforation	Large	32	76.2%	n=29
	Pinhole	2	4.8%	
	Small	8	19.0%	
Location of perforation	Anterior	15	27.8%	n=17
	Inferior (central)	30	55.5%	
	Posterior	9	16.7%	
pre-operative Decible, mean (SD)		17.81 (11.83)		n=0
Pre-operative audiogram	Mild CHL	26	40.0%	n=6
	Moderate CHL	19	29.2%	
	Mixed CHL	15	23.1%	
	SNHL	2	3.1%	
	Normal hearing	3	4.6%	
Pre-operative tympanogram	Type B	59	100.0%	n=12
Pre-operative: Scutum eroded	No	44	97.8%	n=27
	Yes	1	2.2%	
Pre-operative: Ossicles eroded	No	49	98.0%	n=21
	Yes	1	2.0%	
Tympanomastoidectomy	Not done	68	97.1%	n=1
	Canal wall up	2	2.9%	
CT Cholesteatoma	No	69	97.2%	n=0
	Yes	2	2.8%	

Pertaining to comorbidities, only five (7.0%) patients had a documented history of diabetes mellitus (DM) and three (4.2%) patients had a diagnosis of hypertension (HTN). Of note, 25 (35.2%) patients exhibited other comorbidities, whereas the remaining 46 (64.8%) had no additional comorbid conditions (Table 1).

**Figure 1:**
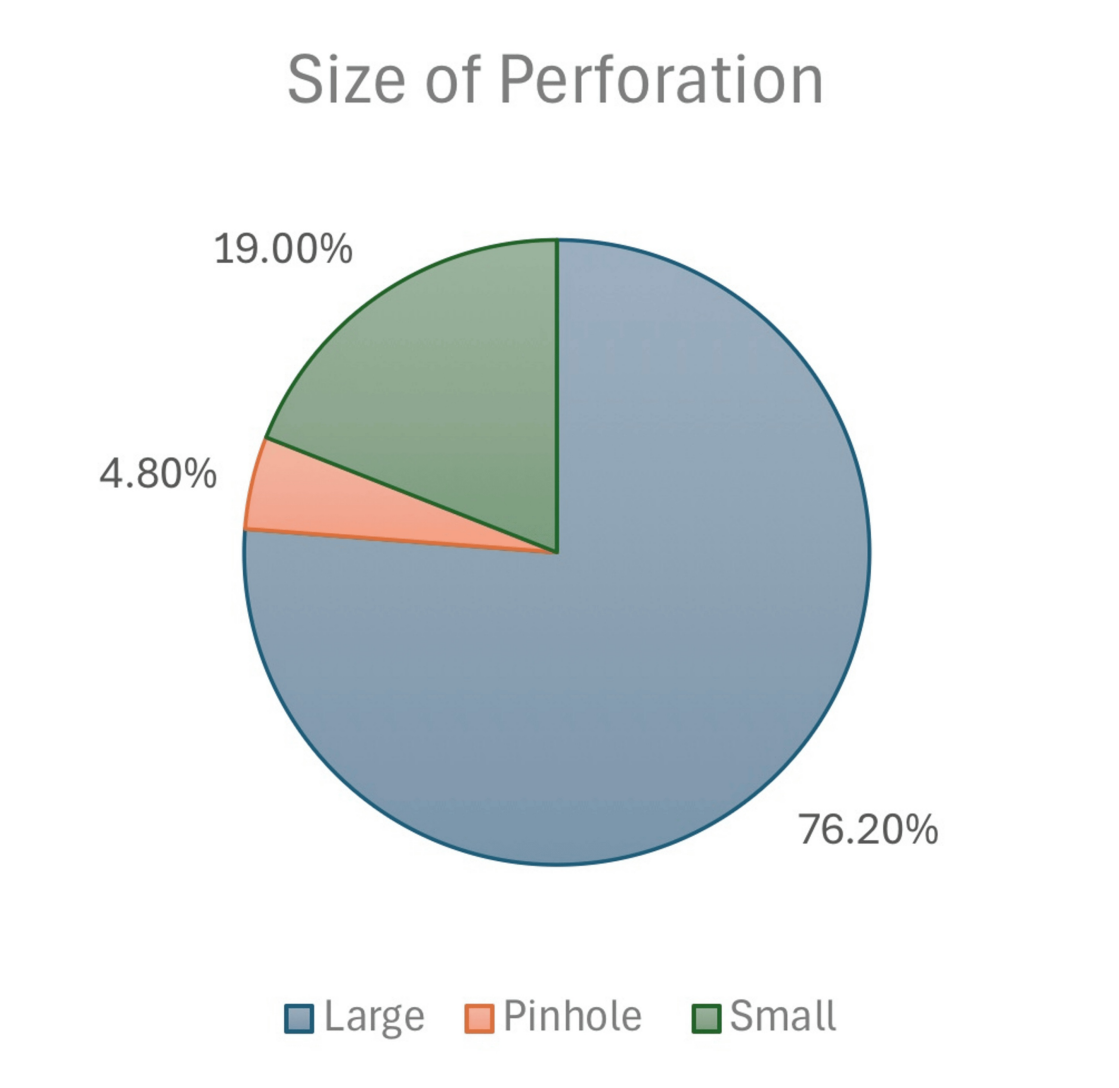
Perforation size classification.

**Figure 2:**
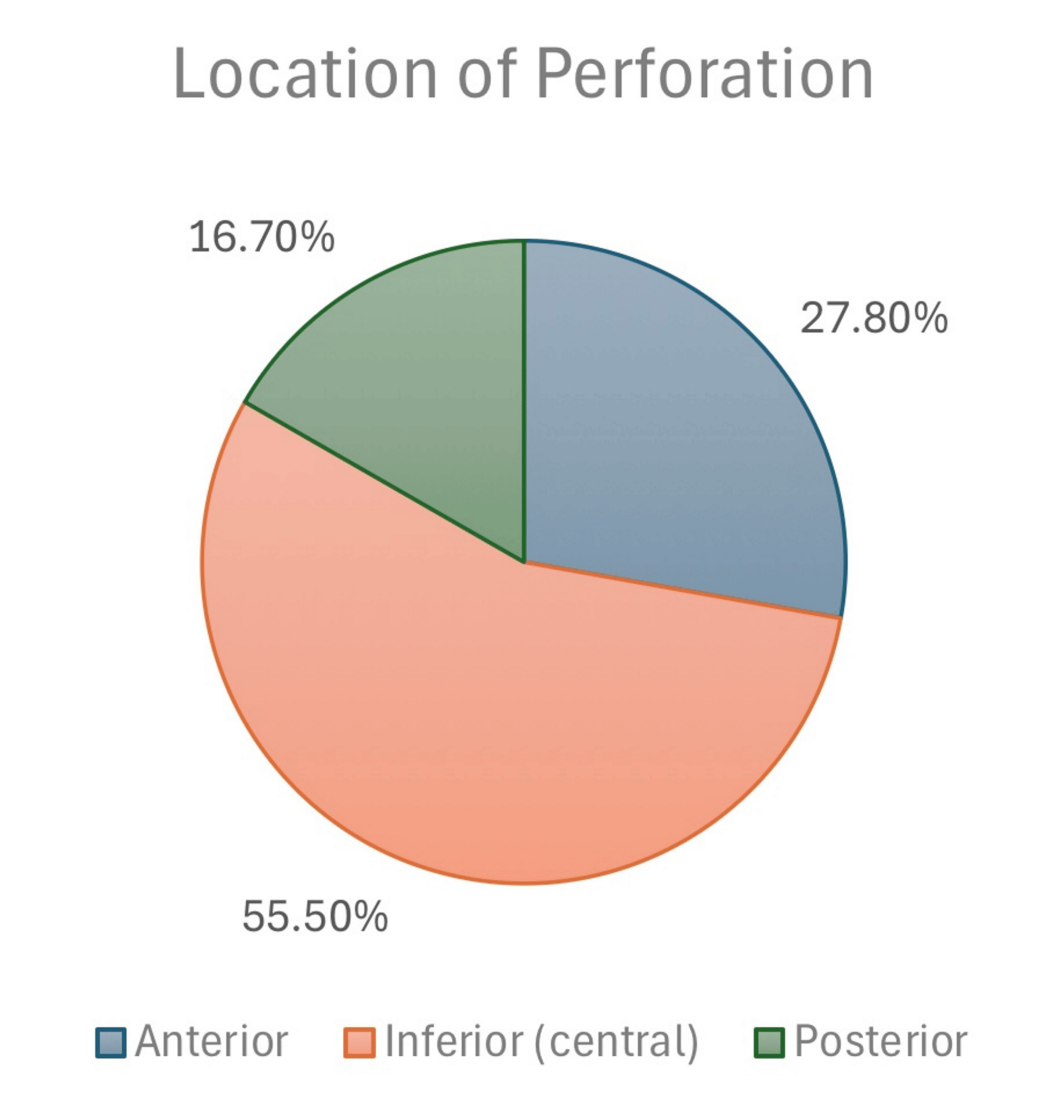
Perforation location classification.

Upon otological examination, it was found that 44 (62.0%) patients had contralateral tympanic membrane perforation. The distribution of ear sites was relatively balanced, with 33 (46.5%) patients presenting with involvement of the left ear. The size of the tympanic membrane perforation varied among the patients, with 54 (76.2%) having a large perforation, 3 (4.8%) having a pinhole-sized perforation, and 13 (19.0%) having a small-sized perforation (Figure 1). Furthermore, the location of the perforation indicated that 19 (27.8%) cases had an anterior perforation, 39 (55.5%) had an inferior (central) perforation, and 12 (16.7%) had a posterior perforation (Table 1, Figure 2).

The pre-operative audiological assessment revealed that among the patients, 28 (40.0%) had mild conductive hearing loss (CHL), 21 (29.2%) had moderate CHL, 16 (23.1%) had mixed CHL, and two (3.1%) had sensorineural hearing loss (SNHL). Pre-operative tympanometry demonstrated that all patients (100.0%) had a Type B tympanogram. Pre-operative decibel levels had a mean of 17.81 dB (SD = 11.83) (Table 1).

Regarding pre-operative conditions, scutum erosion was observed in only one patient (2.2%), while ossicular erosion was present in two patients (2.0%). Additionally, a small subset of patients (n=2, 2.9%) underwent canal wall up tympanomastoidectomy. Furthermore, CT cholesteatoma was not detected in the majority of patients, with 70 patients (97.2%) showing no signs of cholesteatoma (Table 1).

**Figure 3:**
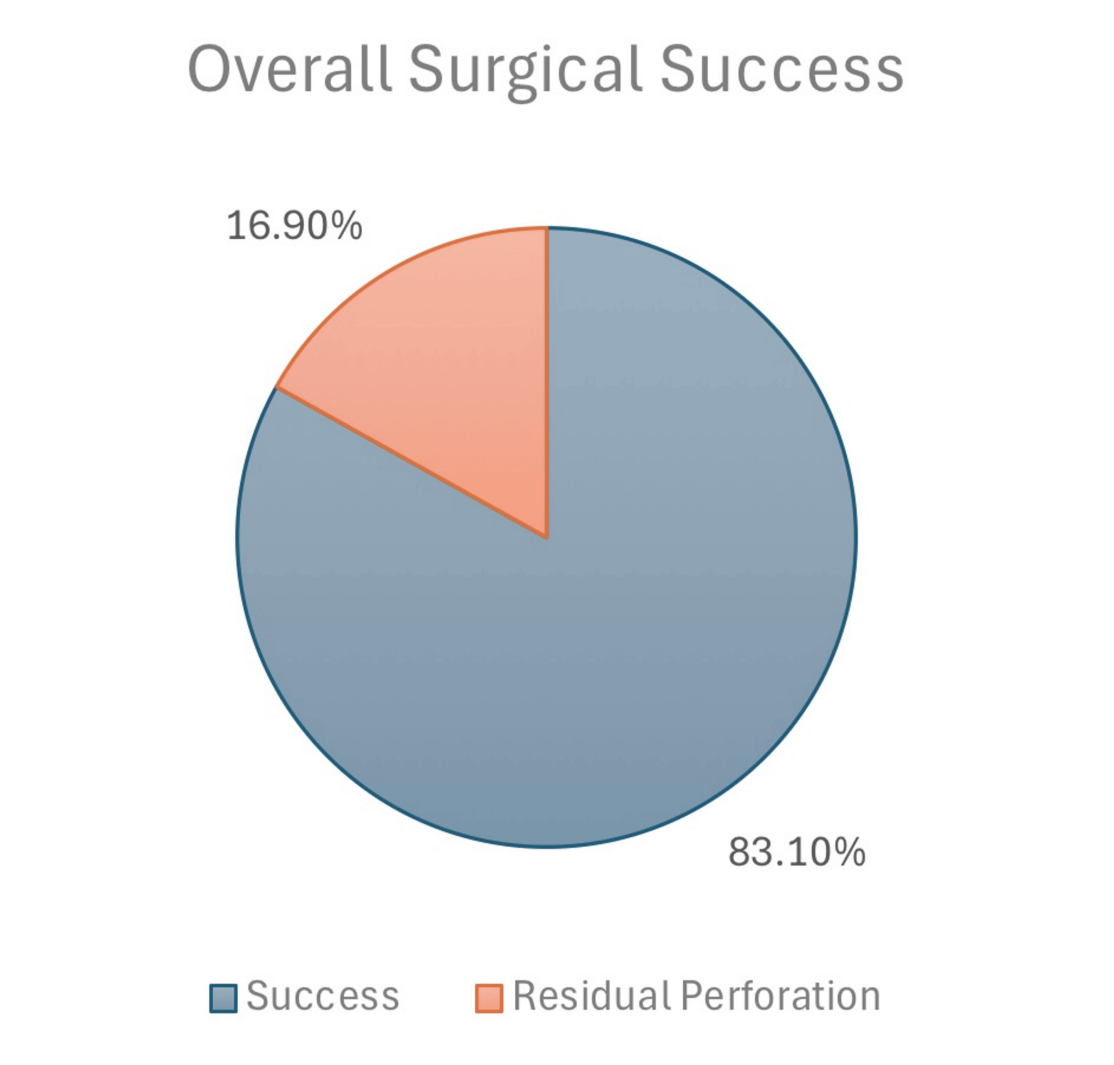
Overall surgical success.

Table 2 focuses on the post-operative outcomes of the tympanoplasty procedures. The mean post-operative decibel was 8.96 (SD = 8.13). The vast majority of patients (59, 83.1%) did not exhibit residual perforation following surgery (Figure 3). Only one (1.4%) patient experienced a recurrence of perforation post-operatively. With regard to the post-operative hearing gap change, 41 (57.9%) patients demonstrated improvement. Revision surgery was required for one (1.4%). patient. The duration of residual perforation, if healed, ranged from 3 to 11 months (interquartile range). The duration until recurrence or residual perforation occurred varied from 0 to 26 months (interquartile range).

**Table 2:** Post-operative outcomes.

Characteristic		N	%	Missing
Post-operative Decible mean (SD)		8.96 (8.13)		
Post-operative residual perforation	No	59	83.1%	n=0
	Yes	12	16.9%	
Post-operative recurrence of perforation	No	70	98.6%	n=0
	Yes	1	1.4%	
Post-op hearing gap change	Improved	22	57.9%	n=33
	No change	16	42.1%	
Revision surgery done	No	70	98.6%	n=0
	Yes	1	1.4%	
Duration of residual if healed in months, Interquartile range (minimum-maximum)		3 (9-11)		
Duration until recurrence/residual perforation in months, Interquartile range (minimum-maximum)		26 (0-26)		

Table 3 provides a comprehensive overview of the post-operative complications observed in the study cohort. The most prevalent complication was infection, encompassing cellulitis, otitis externa, and otitis media, which affected 13 (18.8%) patients. Wound infection was reported in four (5.6%) patients. Other complications, such as recurrent right ear infections with MRSA, growth of normal skin flora in ear culture, graft failure (recurrence), pain and discomfort, and various otological symptoms, were reported in smaller proportions. It is noteworthy that 44 (62.3%) patients did not experience any complications.

**Table 3:** Post-operative complications.

Complication	N	%	Missing
Infection	13	18.8%	n=2
Wound infection	4	5.6%	
Otitis Externa	2	2.8%	
Otitis Media	2	2.8%	
Recurrent right ear infections with MRSA	1	1.4%	
Ear culture: growth of normal skin flora	2	2.8%	
Not specified	2	2.8%	
Graft failure (recurrence)	4	5.8%	
Pain and discomfort	3	4.3%	
Others (e.g., pressure necrosis, auricular numbness, ear fullness, tinnitus, and decreased hearing)	5	7.2%	
Residual perforation	1	1.4%	
None	43	62.3%	

The analysis yielded significant findings, revealing associations between certain variables and post-operative hearing gap change. Specifically, the age at the time of surgery showed a significant association with hearing gap change in the "Improved" group (p < 0.05), whereas no significant association was found in the "No change" group. However, no significant associations were observed between the pre-operative decibel level and hearing gap change in either group, nor between the duration of residual if healed in months and hearing gap change.

On the other hand, a significant association was found between the post-operative decibel level and hearing gap change within the "Improved" group (p < 0.05), while this association did not reach significance in the "No change" group. Additionally, no significant associations were detected between the post-operative follow-up period in months and hearing gap change in either group.

Moreover, the analysis of the data revealed a significant association between post-op residual perforation and post-op hearing gap change (χ^2 = 4.49, df = 1, p = .034). This suggests that there is a relationship between these two variables.

## Discussion

Our study demonstrated a fascia tympanoplasty anatomical success rate of 83.1%, with 57.9% of patients who underwent postoperative audiological assessment showing improved hearing thresholds. This outcome was significantly associated with age and the post-operative decibel levels (mean: 8.96 dB). Additionally, residual perforation was reported in only 12 patients (16.9%) and was significantly associated with reduced functional improvement. The procedure demonstrated a strong safety profile, with 44 patients (62.3%) experiencing no complications, such as ear or wound infections, graft failure, or pain.

Fascia myringoplasty was performed on 71 patients. Functional and anatomical measures were applied in order to assess the success rate. The functional success was associated with hearing threshold improvement. In this study, the mean pre-operative decibel levels were 17.81 dB (SD = 11.83), and the post-operative levels showed improvements with a mean of 8.96 dB (SD = 8.13). Twenty-two patients (57.9%) experienced these improvements. Biswas et al. reported a similar percentage [19].

Several factors may affect the functional outcome, such as age, gender, presence of comorbidities, presence of pre-operative erosions, pre-operative ear status, contralateral tympanic membrane perforation, perforation size, perforation site, and post-operative complications. In our study, age at the time of surgery was significantly associated with hearing threshold improvement. This finding aligns with Joshi et al. [20], who reported that advanced age is a favorable prognostic factor. However, this conclusion contrasts with other reports [21-23], where age was not shown to significantly influence hearing outcomes. Our findings, therefore, contribute to the ongoing debate on age as a prognostic factor in tympanoplasty, suggesting that it may indeed play a role in postoperative functional recovery.

Gender, DM, HTN, pre-operative erosions, site and size of the perforation, and post-operative complications did not show significant associations with audiological improvement in our study. These results are consistent with previous literature [21-24], suggesting that these demographic and clinical variables may not be strong predictors of functional success.

Furthermore, perforation closure was considered an anatomical improvement. The majority of patients (n=59, 83.1%) had successful graft uptake, while 12 patients (16.9%) had post-operative residual perforation. Only two patients had more than one surgery in the same ear, and nine patients had previous surgeries in the contralateral ear. Twenty-seven patients (38.0%) had contralateral tympanic membrane perforation. The most common perforation location was inferior (central) (n=30, 55.5%), followed by anterior and posterior perforations at 27.8% and 16.7%, respectively. Our finding that central perforation was the most common is in agreement with the study by Naderpour et al. [21], while Sharma et al. [25] found anterior perforations to be most frequent and noted their association with complications due to poor vascularity and limited surgical access. These contrasting findings suggest that perforation site distribution may vary geographically or demographically and that anterior perforations might present a greater surgical challenge regardless of frequency.

Large perforations were the most common size in our sample, followed by small and pinhole perforations at 76.2%, 19.0%, and 4.8%, respectively. These results mirror those of other studies [22,25,26], supporting the general observation that large perforations are often the most encountered in clinical practice. In contrast, Pillai et al. reported medium-sized perforations as the most prevalent [27], suggesting that classification thresholds or referral patterns may influence these proportions. Despite the frequency of large perforations, our data did not show a significant effect of perforation size or site on graft uptake. This contrasts with findings by Das et al. [26], who reported that larger perforation size adversely affected outcomes, although site was not statistically significant. Similarly, Sharma et al. [25] found both size and site to be significant predictors of graft failure, particularly in anterior and large-sized perforations. These inconsistencies across studies may be due to methodological differences or variations in surgical technique and patient selection.

Interestingly, the presence of contralateral perforation, the site and size of perforation, and other demographics such as sex and pre-operative erosions did not have significant effects on graft uptake in our study. This observation is reinforced by prior studies that similarly reported no significant associations between age, gender, and surgical outcomes [21,23]. However, contrasting evidence from another tertiary center reported that contralateral ear pathology negatively influenced surgical prognosis, with 88.0% of patients having normal contralateral ears in that study [22].

Further, there are disparities in the literature regarding the impact of the site and size of perforation on graft uptake. In a study with similar results to our study, where the large size and central perforation site were the most frequent, Das et al. reported that perforation size significantly influenced the procedure outcome, whereas the perforation site failed to be a statistically significant factor [26]. In a study that included 53 patients, the most common site was anterior perforation, and the commonest perforation size was large. The study resulted in a significant association between the perforation size and graft failure. Also, the perforation site significantly influenced graft re-perforation [25]. A similar significant impact of the size and site on hearing outcome was reported in a different study with the medium size and the anterior site being the commonest [27,28]. These findings highlight a lack of consensus in the literature regarding the prognostic value of perforation characteristics. Our results contribute to this debate by suggesting that, at least in this cohort, these factors did not significantly alter outcomes.

Furthermore, our study showed a statistically significant association between the absence of residual perforation and post-operative hearing gap improvement, indicating that anatomical closure of the tympanic membrane directly contributes to functional recovery. This observation aligns with the findings of Batni et al. [29], who similarly concluded that intact graft uptake plays a critical role in audiological outcomes. This emphasizes the dual importance of achieving both anatomical and functional success in tympanoplasty.

Finally, in a meta-analysis conducted to determine independent variables affecting the efficacy of type I tympanoplasty, a total of 214 studies were reviewed with an average success rate of 88.6%. Within this group, studies employing fascia grafts reported a success rate of 88.0% [30], which is slightly higher than the rate observed in our study. Notably, variables with favorable significant effects included adult age, small perforation size, and the use of cartilage grafts. In contrast, other factors such as surgical technique, follow-up duration, perforation cause and site, and ear status were not found to significantly affect outcomes. This meta-analysis provides a broad context in which to interpret our results, suggesting that while our outcomes are consistent with global trends, individual study variations, including sample characteristics and surgical methods, can influence success rates.

This retrospective study has several limitations that should be considered when interpreting the findings. Firstly, the study design is inherently prone to bias, as it relies on existing medical records that may be incomplete or inaccurate. Secondly, the study was conducted at a single tertiary care center, which may limit the generalizability of the results to other settings or populations. Thirdly, the relatively small sample size may have affected the statistical power and precision of the findings. Moreover, only 38 of the 71 patients underwent post-operative audiological assessment, resulting in incomplete follow-up data that may have introduced attrition bias and limited the strength of conclusions related to hearing outcomes. A formal comparison of baseline characteristics between patients who completed follow-up and those who did not was not performed, which further limits interpretation. Additionally, important prognostic variables such as Eustachian tube function and smoking status, both known predictors of tympanoplasty success-were not captured due to limitations in documentation. The absence of these potential confounding variables restricts the depth of the analysis. Lastly, the lack of a control group prevents the establishment of causal relationships or attribution of outcomes solely to the fascia tympanoplasty procedure.

## Conclusions

In conclusion, this study evaluated the outcome, efficacy, and safety of fascia tympanoplasty procedures for repairing perforated tympanic membranes. The procedure demonstrated a good success rate, with low complication rates and moderate audiological improvement. Age was identified as a significant factor influencing hearing outcomes. Most patients had successful graft uptake, with only some patients experiencing post-operative residual perforation. Various factors such as gender, comorbidities, and post-operative complications did not significantly impact audiological improvement. However, the absence of residual perforation was associated with better post-operative hearing function. Overall, fascia tympanoplasty demonstrated a good success rate with favorable anatomical and functional outcomes, and factors such as age and perforation closure played significant roles in surgical success. Further research with a larger sample size is needed to validate these findings.
